# Metabolic adaptation to hypoxia: do worms and cancer cells share common metabolic responses to hypoxic stress?

**DOI:** 10.1038/s41418-021-00741-y

**Published:** 2021-02-12

**Authors:** Ralf Baumeister, Coleen T. Murphy, Thomas Heimbucher

**Affiliations:** 1 Bioinformatics and Molecular Genetics, Institute of Biology 3, Faculty of Biology, University of Freiburg, Freiburg, Baden-Wuerttemberg Germany; 2 Center for Biochemistry and Molecular Cell Research (ZBMZ), Faculty of Medicine, University of Freiburg, Freiburg, Baden-Wuerttemberg Germany; 3 Signalling Research Centres BIOSS and CIBSS, University of Freiburg, Freiburg, Baden-Wuerttemberg Germany; 4 Lewis-Sigler Institute for Integrative Genomics, Princeton University, Princeton, NJ USA; 5 Department of Molecular Biology, Princeton University, Princeton, NJ USA

**Keywords:** Fatty acids, Gene expression

The survival of metazoan species is dependent on an intricate balance of oxygen supply and demand. In humans this balance is disturbed during age-related diseases including disorders of the cardiovascular system and cancer. Rapidly growing cancer cells become hypoxic in the absence of a functional vascular system. The resulting oxygen and nutrient stress causes rewiring of essential metabolic pathways to ensure survival of cells in hypoxic tumor areas, which often contain the most malignant tumor cells. Thus, deciphering metabolic mechanisms keeping hypoxic cells alive is critical for improving cancer therapies.

In a recent study, the nematode *C. elegans* has been utilized as a model to analyze hypoxic adaptation through changes in metabolism [1]. *C. elegans* is a suitable model organism to dissect mechanisms conferring hypoxic adaptation. As a soil-living nematode, it can survive in low oxygen environments, even in anoxic conditions, by entering suspended animation [2]. Suspended animation is a reversible, hypometabolic state, ideal to investigate metabolic changes of a hypoxic organism.

**Fig. 1:**
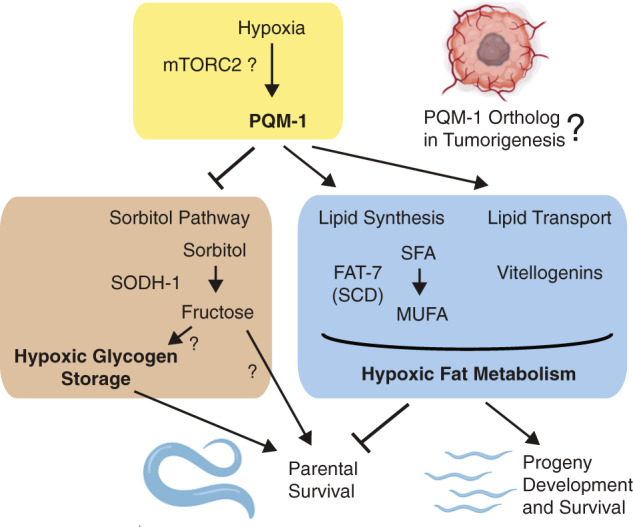
The zinc-finger transcription factor PQM-1 regulates the sorbitol pathway (polyol pathway) and lipid metabolism in hypoxia. PQM-1 is activated in response to hypoxic stress and represses the expression of the sorbitol dehydrogenase (SODH-1) and glycogen levels in worms. SODH-1 enzymatic activity converts sorbitol, the sugar alcohol form of glucose, into fructose. PQM-1 promotes de novo lipid synthesis in hypoxia via expression of the Δ9-fatty-acid desaturase FAT-7 (SCD homolog) and positively regulates lipid transport to embryos through vitellogenin expression. PQM-1-mediated hypoxic activation of lipid metabolism limits parental survival, but is beneficial for progeny development and survival under hypoxic stress. Future studies are needed to identify a functional mammalian equivalent of PQM-1 downstream of mTORC2 signaling, and to dissect its role for tumorigenesis in hypoxic microenvironments. mTORC2 mammalian target of rapamycin complex 2, SCD stearoyl-CoA desaturase, SFA saturated fatty acid: stearic acid, MUFA mono-unsaturated fatty acid: oleic acid.

In the recent *C. elegans* study, the C2H2 zinc-finger (ZF) transcription factor PQM-1 was identified as a regulator of lipid and carbohydrate metabolism and nematodes’ hypoxic survival [1]. Loss of PQM-1 activity extended survival of hypoxic worms, which correlated with metabolic changes. Hypoxic *pqm-1* loss-of-function mutants displayed decreased fat levels and increased amount of glycogen, indicating that PQM-1 activity likely normally promotes lipid accumulation, however, represses glycogen levels in hypoxia (Fig. 1). Thus, downregulation or loss of PQM-1 activity appears to regulate a metabolic switch from fat to glycogen, which is associated with an improved survival of worms in hypoxic stress. Previous findings demonstrate that the ability of *C. elegans* to store carbohydrates such as glycogen correlates with worms’ survival in the absence of oxygen [3]. In analogy, cancer cells can increase their glycogen storage in response to hypoxia to maintain cell viability and proliferation in nutrient and oxygen depleted microenvironments [4]. Hypoxic tumor cells are largely dependent on fermentative glucose metabolism for ATP production (Warburg effect) and can metabolize intracellular glycogen without requiring oxygen, when glucose availability is limited. These findings point to an important and evolutionarily conserved role of glycogen utilization for survival of hypoxic cells across species.

PQM-1 transcriptional targets involved in carbohydrate metabolism were identified by a global gene expression analysis [1]. This analysis revealed an upregulation of the sorbitol dehydrogenase-1 *sodh-1* in *pqm-1* mutants under hypoxia, suggesting that PQM-1 normally suppresses *sodh-1* expression (Fig. 1). SODH-1 enzymatic activity converts sorbitol, the sugar alcohol form of glucose, into fructose. Elevated SODH-1 expression contributed to the extended hypoxic survival of *pqm-1* mutants. Why should an increased SODH-1 function, which likely results in higher fructose levels, be beneficial for an organism suffering from oxygen deprivation? Utilizing fructose instead of glucose for ATP production during hypoxia induces metabolic adaptations in naked mole-rats [5] that live in subterranean burrow systems under low oxygen conditions. In naked mole-rats the metabolization of fructose is able to bypass a feedback inhibition in glycolysis, which usually limits the glycolytic flux. This enables a fructose-based metabolism to safeguard ATP in the brain and other vital organs when oxygen is limited. It is not known yet whether metabolic rewiring toward fructose metabolism is critical for survival of *C. elegans* in hypoxia, but increased fructose intake is associated with tumor growth. Interestingly, hypoxia-adapted glioblastoma cells upregulate both sorbitol and fructose levels; in fact, fructose levels became massively elevated (~80-fold) [6]. In addition, an activation of the sorbitol (polyol) pathway has been linked to a reduction of necrosis in hypoxia-preconditioned pheochromocytoma (PC12) cells transferred to acute anoxia [7]. These studies indicate an upregulation of an alternative glucose metabolic route to ensure energy production and survival of hypoxic cells.

*C. elegans* PQM-1 has recently been identified as a transcriptional effector of mTORC2 signaling [8]. The mTORC2 pathway plays a critical role in regulating glucose and lipid metabolism [9] and, when hyperactive, can promote tumorigenesis in various cancer models. In *C. elegans*, mTORC2 signaling appears to negatively regulate PQM-1 nuclear localization and activity through SGK-1 (serum and glucocorticoid-regulated kinase-1) [8]. Thus, one might speculate that hyperactivation of mTORC2 in mammalian hypoxic tumor cells also should inactivate a PQM-1 ortholog. If the mTORC2/PQM-1 signaling axis is conserved in mammals, such an inactivation of a mammalian PQM-1 ortholog could result in a similar fat-to-glycogen metabolic switch, which was observed in hypoxic *C. elegans pqm-1* mutants. But so far, a PQM-1 ortholog with similar functions remains to be identified. PQM-1 is member of the C2H2-ZF family that has undergone extensive expansion through repeated duplication and functional diversification in vertebrate linages, especially in primates and humans. C2H2-ZF proteins can promote cancer progression, but also act as tumor suppressors [10]. A potential candidate for a human homolog of PQM-1 is the C2H2-ZF transcription factor ZNF395, which is induced in a lymphoma cell line, and in neuroblastomas and glioblastomas in response to hypoxia [10, 11]. In skin cancer and glioblastoma cell lines, ZNF395 promotes cancer-associated gene expression and inflammation [11], whereas in liver cancer cell lines it appears to inhibit cell migration and invasion [12]. Thus, its function in cancer progression seems to depend on the cancer type and on the degree of oxygen availability for tumor cells. A putative metabolic impact of ZNF395 on cancer development should therefore be analyzed.

In the recent *C. elegans* study, an interesting mechanistic link between PQM-1 and the maintenance of lipid levels under hypoxia was found, since PQM-1 was shown to regulate the expression of the Δ9-fatty-acid desaturase FAT-7 (Fig. 1). FAT-7 is a homolog of mammalian stearoyl-CoA desaturases (SCDs), which act as rate-limiting enzymes for de novo lipogenesis. SCDs catalyze the Δ9-desaturation of saturated fatty acyl-CoAs, preferentially stearoyl- and palmitoyl-CoA, and require molecular oxygen for their enzymatic reaction. In *C. elegans* loss of either PQM-1 or FAT-7 activity reduced oxygen consumption rates in worms [1]. In comparison, pharmacological inhibition of SCDs in tumor cell lines decreased respiration [13]. At the first glance, PQM-1 and FAT-7 dependent pro-lipogenic responses in worms seem to be an evolutionary disadvantage, since their metabolic functions increase oxygen consumption, and thus compromise survival in hypoxia. However, PQM-1 also fuels reproduction of worms by promoting lipid transport to worm embryos and their subsequent development and survival under hypoxic stress (Fig. 1). Such stress response mechanisms benefiting the next generation and the survival of a species could have developed early in evolution, but might still control lipid-related metabolic responses favoring growth and proliferation in mammals. Tumor cells are heavily dependent on SCD-mediated de novo synthesis of unsaturated fatty acids for their rapid growth, although certain tumors are able to bypass SCD activity by scavenging unsaturated fatty acids from their microenvironments, when oxygen is depleted. Mouse embryonic fibroblasts with constitutively active mTOR signaling undergo cell death when grown in lipid- and oxygen-deprived conditions due to an impaired function of SCDs which causes a deficiency in unsaturated lipid pools [14]. Thus, SCD levels and oxygen availability for their enzymatic reaction to generate unsaturated lipids are critical determinants for tumor growth and survival. In addition to its role in SCD-mediated de novo lipogenesis, PQM-1 is involved in lipid transport in nematodes via coordinating vitellogenin expression (Fig. 1) [1, 8]. Similarly, in humans, the C2H2*-*ZF transcription factor ZNF202 regulates the expression of components for lipoprotein particles (apolipoproteins), which transport lipids through the circulatory system [15]. ZNF202 has been identified as an unfavorable prognostic marker for liver and renal cancer, however, its function has not been analyzed in the context of hypoxia or mTOR signaling yet. Future studies might identify a functional mammalian PQM-1 homolog downstream of mTORC2 signaling as a drug target to manipulate lipid-metabolic adjustments affecting survival responses of cancer cells in hypoxic microenvironments.
